# Identification of Thioflavin T Binding Modes to DNA: A Structure-Specific Molecular Probe for Lasing Applications

**DOI:** 10.1021/acs.jpclett.1c01254

**Published:** 2021-06-03

**Authors:** P. Hanczyc, P. Rajchel-Mieldzioć, B. Feng, P. Fita

**Affiliations:** † Institute of Experimental Physics, Faculty of Physics, University of Warsaw, Pasteura 5, 02-093 Warsaw, Poland; ‡ Department of Chemistry and Chemical Engineering, Chalmers University of Technology, 412 96 Gothenburg, Sweden

## Abstract

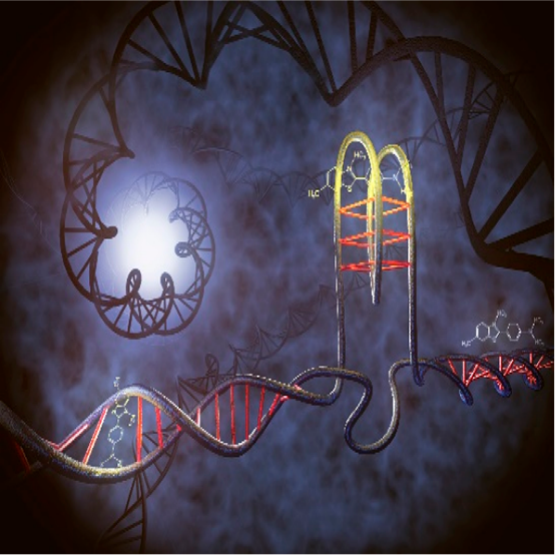

The binding mechanism of thioflavin T (ThT) to DNA was studied using polarized light spectroscopy and fluorescence-based techniques in solutions and in solid films. Linear dichroism measurements showed that ThT binds to DNA duplex by intercalation. Time-resolved fluorescence studies revealed a second binding mode which is the external binding to the DNA phosphate groups. Both binding modes represent the nonspecific type of interactions. The studies were complemented with the analysis of short oligonucleotides having DNA cavities. The results indicate that the interplay between three binding modes—intercalation, external binding, and binding inside DNA cavities—determines the effective fluorescence quantum yield of the dye in the DNA structures. External binding was found to be responsible for fluorescence quenching because of energy transfer between intercalated and externally bound molecules. Finally, amplified spontaneous emission (ASE) was successfully generated in the ThT-stained films and used for detecting different DNA structures. ASE measurements show that ThT-stained DNA structures can be used for designing bioderived microlasers.

Thioflavin T is a small organic fluorophore that belongs to the group of molecular rotors.^1,2^ In solution, it has a low fluorescence quantum yield because of an ultrafast torsional motion that drives nonradiative deactivation via twisted intramolecular charge transfer (TICT).^3^ When internal rotation of its molecular segments is inhibited, there is a hundreds-fold increase of the fluorescence signal.^4^ It was found that ThT emission is highly sensitive to the local structure of surrounding biomolecules and their microenvironment.^5−7^ That makes ThT a fluorophore widely used in biomedical research,^8^ primarily to detect amyloid protein fibrils that are linked to neurodegenerative diseases.^9^ Because ThT is a well-accepted histological dye, there is also a growing interest in using it for sensing various biologically relevant structures.^10−12^

Recently, interactions of ThT with DNA have been reported.^13−18^ It was found that ThT exhibits a moderate emission enhancement in the presence of single- and double-stranded DNA, whereas a strong fluorescence could be detected upon ThT insertion into DNA cavities such as internal gaps or mismatch sites.^19^ Among the specific DNA cavities there are a few of high biological significance, including i-motifs and G-quadruplexes (G4).^20,21^ Both DNA structures are formed by stacking of Hoogsteen base pairs of cytosines or guanines, respectively. Their genetic relevance found in the expression of tumor cells, gene regulation, or cell division^22−24^ stimulated an interest in finding selective biomarkers for these motifs. A very promising candidate for such a biomarker is ThT, because it exhibits a remarkable fluorescence enhancement in the presence of both the i-motif and G-quadruplex structures (G4).^25−27^

In this Letter, we examine the ThT interactions with four DNA samples: a natural long DNA duplex from calf thymus, and 28 bases long synthetic single- and double-stranded DNA, in which one of the single-stranded oligonucleotides mimics the classical DNA strand and the other, G-rich oligonucleotide, forms the G-quadruplex structure (sequences are presented in the Supporting Information). The last examined structure was a synthetic duplex with mismatched base pairs creating an internal loop formed upon hybridization of two complementary single strands (Figure 1).^28^

**Figure 1:**
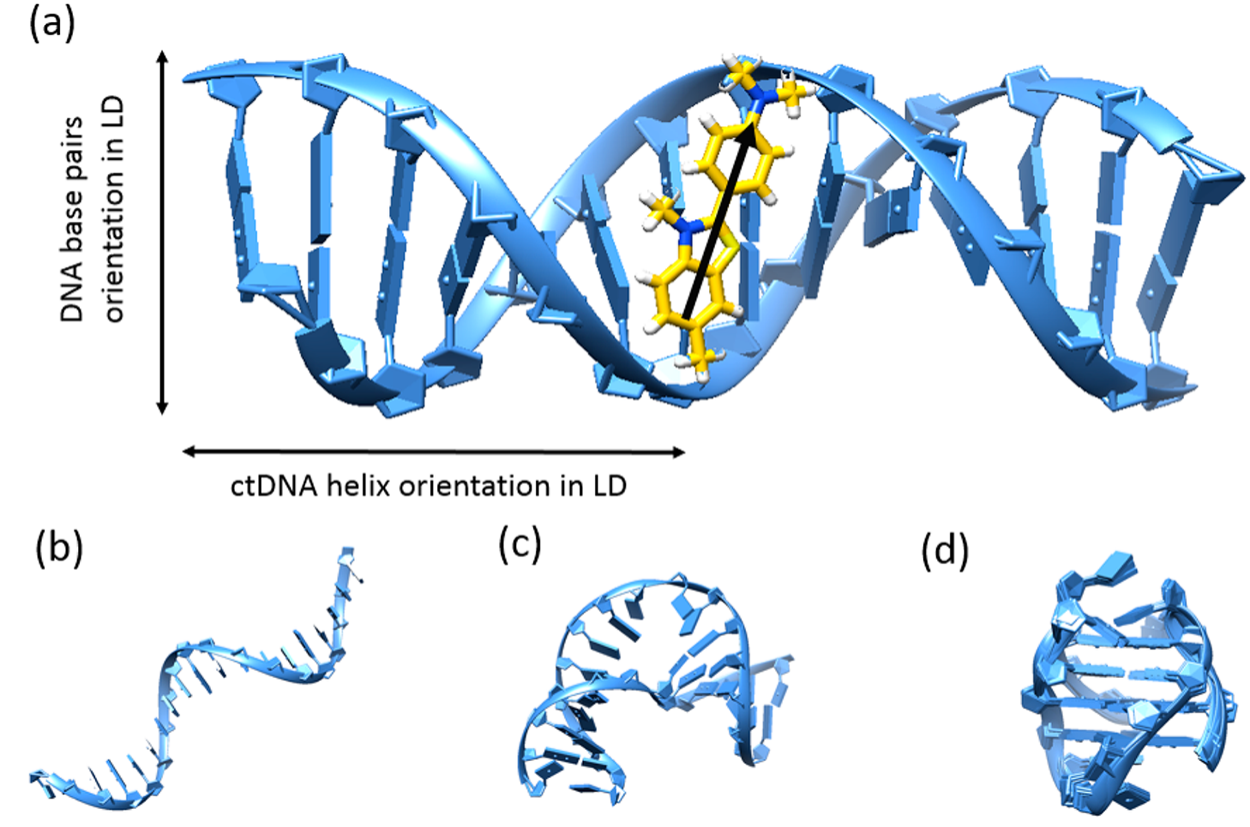
(a) ctDNA duplex with thioflavin T (yellow structure) intercalated between DNA strands with the orientation of the transition dipole moment marked with the black arrow, (b) single-stranded DNA (ssDNA), (c) double-stranded (dsDNA) with the mismatched base pairs forming the internal loop, and (d) G-quadruplex G4 DNA.

The DNA samples were examined in solutions and in solid DNA-based films using linear dichroism (LD), time-correlated single-photon counting (TCSPC), and amplified spontaneous emission (ASE) methods (a schematic illustration of the setup for ASE measurements in ThT-stained DNAs is shown in Figure S1). LD was used to study binding modes of ThT to calf thymus DNA in solution. The TCSPC technique was used to record time-resolved fluorescence of ThT-stained DNA samples both in the liquid phase as well as in the solid state. ASE was applied to solid films in order to verify whether this technique can differentiate between various DNA structures. To the best of our knowledge, a comprehensive analysis of various ThT-stained DNA structures using a versatile toolkit for determining the various ThT binding modes has not been performed yet.

Considering the LD and TCSPC results, we found that ThT can bind to DNA in three modes: specific binding to DNA cavities, intercalation between DNA bases, and external binding to the DNA phosphate groups. ThT bound in each mode has a different yield of fluorescence. The emitting ThT species are located in DNA cavities and intercalation sites, whereas ThT molecules externally bound to the DNA phosphate groups quench the fluorescence of the former.

The ThT-stained DNA films were analyzed using ASE. ASE spectra, full width at half-maximum (fwhm), and thresholds were shown to be parameters sensitive to the specific DNA structure. The DNA structural richness can be utilized in self-switchable microlasers^29^ and for detection of cancerous DNA motifs.^30^ In the presented system we combined the structural richness of DNA with the discovery of ASE in the ThT dye.

In general, three types of nonspecific binding of dye molecules to DNA can be distinguished: intercalation whereby dye molecules slide inside the DNA helix between the base pairs, groove binding whereby the dye is accommodated outside the double-helix in the minor or major groove of the DNA, and external binding whereby the dye interacts with the phosphate groups of the nucleotides.^31^ The binding type can be determined using linear dichroism (LD) spectroscopy (details of the LD measurements are given in the Supporting Information). The LD spectrum obtained for a DNA–dye complex oriented in shear flow provides information on the orientation of bound dye molecules relative to the DNA helix axis (Figure 1a). Nonzero values of LD are obtained for intercalation and groove binding, whereas external binding and noninteracting dye molecules should exhibit zero LD. From the four DNA molecules examined in this Letter (Figure 1), ctDNA, ssDNA, dsDNA with the internal loop, and G4 DNA, only the ctDNA molecule is of sufficient length be oriented in the flow solution.^32^ Thus, at first the ThT-stained ctDNA was analyzed in the context of the dye binding modes.

The negative LD peak (Figure 2a) at 260 nm arises from the dipole moments of nucleotides stacked perpendicular to the helix axis, which in turn aligns with the shear flow. The LD contributions above 300 nm, where DNA does not absorb, stem from ThT. The negative LD sign in the main absorption band of the dye (maximum at 445 nm) indicates that the long axis of the ThT chromophore is overall aligned perpendicular to the helix axis and ThT intercalates between the DNA base pairs. To know the exact angle of ThT relative to the DNA helix axis, the orientation factor (*S*) for DNA bases was first calculated using equations described in the Supporting Information, and it was equal to 0.1 at 260 nm. The same *S* factor was assumed for bound ThT, and knowing that LD^r^ = LD/A_iso_ = −0.1 of bound ThT at 445 nm, the exact angle was calculated as α_dye_= 70° relative to the helix axis.

**Figure 2:**
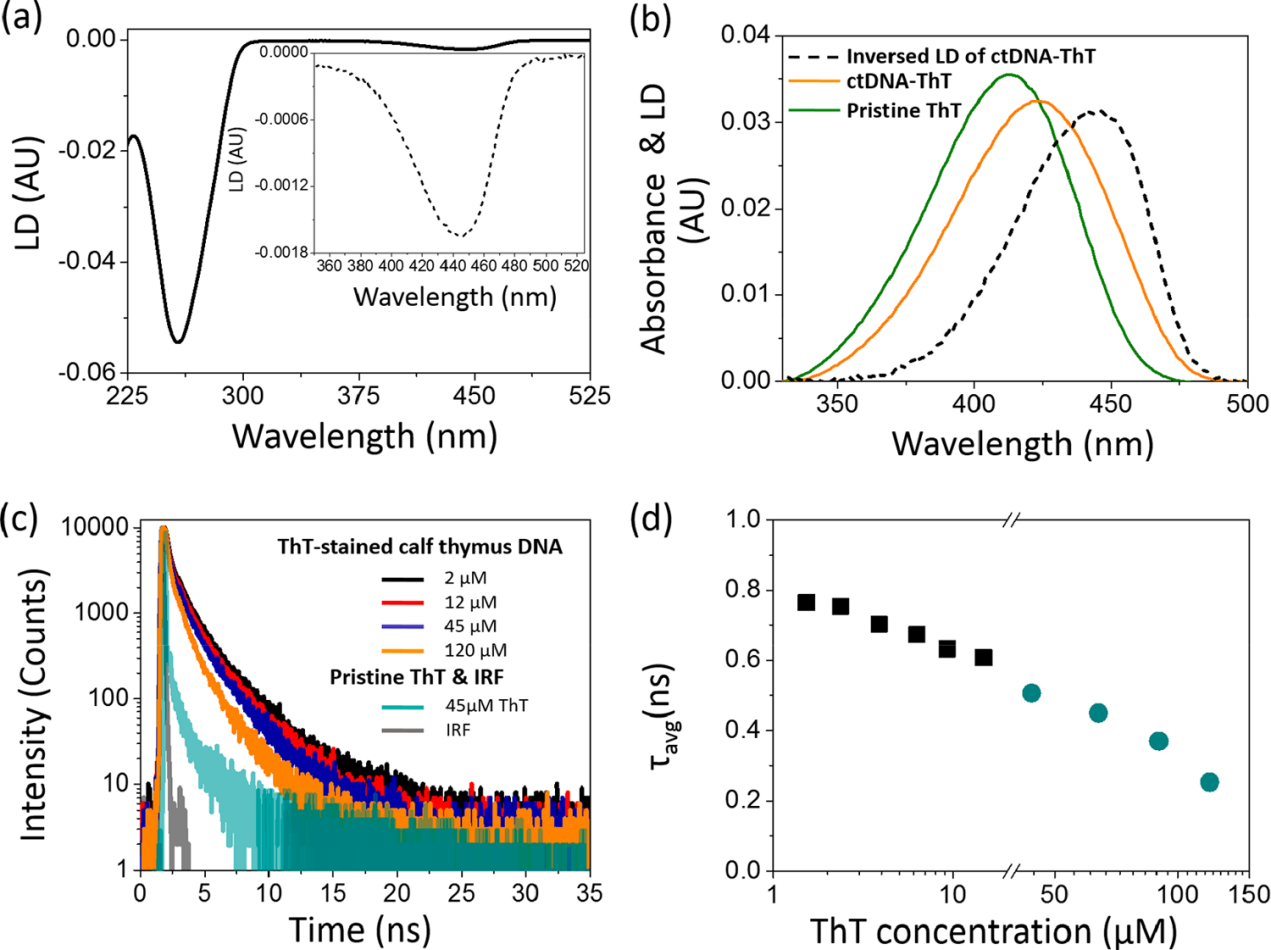
(a) Linear dichroism spectrum of ctDNA with ThT ([ThT] = 9 μM). Inset shows the LD spectrum enlarged in the spectral range of the ThT main absorption band. (b) 1 mm absorption spectra of pure ThT (9 μM in water, green), ctDNA-ThT mixture (identical to panel a, orange) and the LD spectrum of the ctDNA-ThT mixture multiplied by −20 for the sake of comparison (black dashed curve). (c) Examples of fluorescence decays of ctDNA-ThT mixtures recorded at various ThT concentrations, 2 μM (black), 12 μM (red), 45 μM (dark blue), and 120 μM (orange), and of pristine ThT in a PBS solution (cyan). Samples were excited at 405 nm (3.06 eV). The instrumental response function (IRF) is shown in gray. (d) The average ThT fluorescence lifetimes calculated on the basis of fitting the three-exponential function to the fluorescence decays recorded for various ThT concentrations in the range of 2–120 μM.

In Figure 2b, 1 mm absorption spectra of pure aqueous ThT and a ThT–DNA mixture (containing both free and bound ThT molecules) are complemented with the absolute value of the ThT LD spectrum. The interaction of ThT with DNA leads to a bathochromic shift of the absorption spectrum with the maximum shifted to 422 nm as compared to 411 nm recorded for the free dye in bulk water. A previous photophysical investigation of ThT in solvents of different polarity has demonstrated that the bathochromic shift of the absorption spectrum of ThT is caused by reduced polarity.^33^ The red-shift of the LD spectrum can be explained by the electrostatic attraction of ThT molecules to the intercalation sites which results in a change of their environment from polar water to nonpolar space between the base pairs.

Because free ThT lacks LD signal, the absolute value of the ThT LD peaks provides the shape of the absorption spectrum of ThT molecules bound to DNA. Therefore, the absorbance spectrum of a ThT–DNA mixture can be resolved into pure and bound ThT. Comparison of the LD spectrum that represents only the intercalated form of ThT and the total absorption spectrum of the DNA–dye mixture revealed that 38% of the dye molecules were intercalated between the DNA base pairs. In the intercalation site the dye rotation should be inhibited, and in the case of ThT, the inhibition of the ring rotation is usually associated with a sizable fluorescence enhancement.^2^ Intriguingly, only scarce emission was observed for ThT doped to ctDNA (Figure S2a).

To investigate the possible mechanism responsible for the relatively low fluorescence quantum yield of ThT in the presence of ctDNA, time-resolved experiments were performed. Figure 2c shows selected ThT fluorescence decays recorded in solution for a constant ctDNA concentration with the dye concentration increasing from 2 to 120 μM (the decays in a short time range, up to 3 ns, are shown in Figure S2b). The decays were fitted with a three-exponential function, and the average lifetimes were calculated for a set of ThT concentrations. A significant shortening of the average fluorescence lifetime is observed with increasing dye concentration (Figure 2d). At low ThT concentrations it decreases linearly with the increasing concentration, and above approximately 15 μM ThT this dependence slows down.

In the case of free organic fluorophores in solutions, the fluorescence quenching due to FRET usually occurs at millimolar concentrations because of the decreasing interspace distance between dye molecules.^34^ Here, in the presence of ctDNA, quenching of ThT fluorescence was observed already at the micromolar concentration range, at least an order of magnitude lower than for the free dye in solution. This indicates that besides intercalation, ThT must bind to DNA preserving some degree of ring rotation that allows deactivation through twisted intramolecular charge transfer (TICT). In such a case, homo-FRET may occur between the strongly emissive intercalated ThT molecules and a population of dye molecules that we hypothesize bind to the exterior of the DNA helix. We emphasize that externally bound ThT molecules are located at a much shorter distance from the intercalated ones than unbound, free-floating molecules. The externally bound molecules preserve some degree of flexibility and undergo nonradiative deactivation at a rate which is relatively fast in comparison to that of the intercalated molecules. Thus, they act as acceptors of the excitation energy from the fluorescent species,^35^ and the TICT-related nonradiative deactivation of the externally bound molecules results in the low effective fluorescence quantum yield of ThT in the presence of the ctDNA duplex (Figure S2a).

The most probable binding mode responsible for the observed fluorescence quenching is external binding to the phosphate groups located at the backbone of the DNA helix. In order to examine whether ThT can bind externally to the DNA helix, fluorescence of ThT mixed with single-stranded DNA (ssDNA) oligonucleotide was investigated. In the case of ssDNA, dye binding between DNA strands at the intercalation sites is not possible because there is only one strand.

Figure 3 shows fluorescence decays of ThT-stained ssDNA in solution and in a drop-cast film. They are very similar to each other and significantly slower than the fluorescence decays recorded for pristine ThT either in solution or in the solid phase. This indicates that the mechanism responsible for the decays’ slowdown is the same in both environments. Even if the effect in the film could be caused by a reduction of the free volume available for ThT molecules and their immobilization in a rigid environment, it is unlikely that fluorescence decays of such a similar character are caused by different mechanisms. Moreover, solidification of the environment alone exerts a much weaker effect on the fluorescence decay of ThT, as shown by the fluorescence decay of pristine ThT in the film. Thus, an additional mechanism must be active in the films made of ThT and ssDNA mixtures, which proves that ThT indeed binds to ssDNA in both liquid and solid environments.

**Figure 3:**
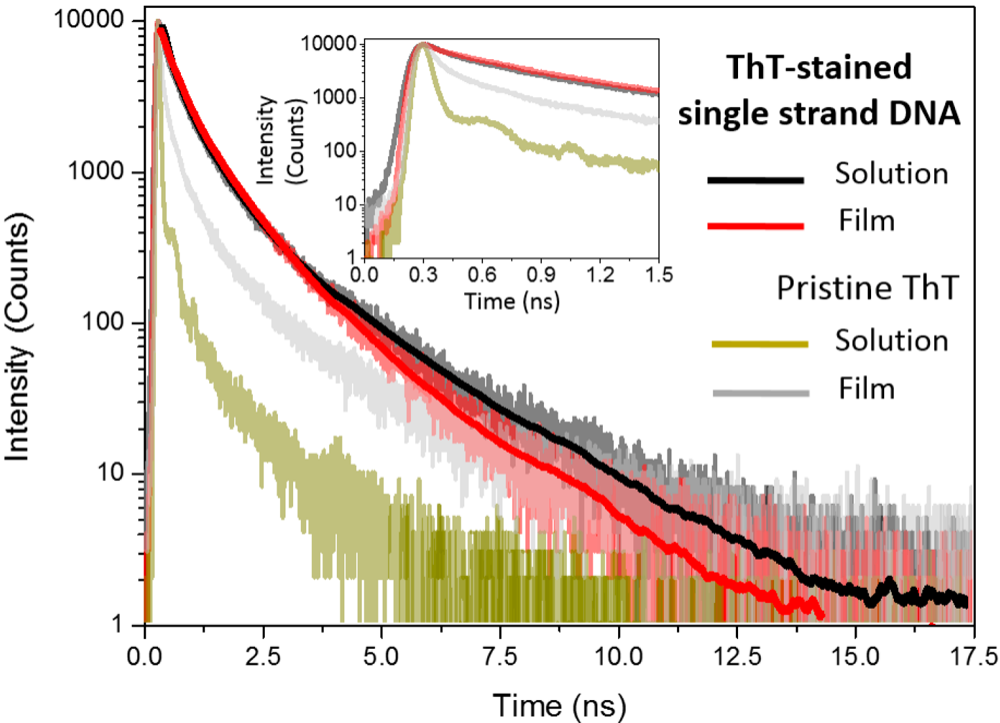
ThT fluorescence decays in the presence of ssDNA in a solution (black) and in a drop-cast film (red). For reference, the fluorescence decay of pristine ThT in a solution is shown in green, and that of solid ThT deposited on a glass slide by drop casting is in gray. The inset shows decays in the time scale to 1.5 ns. Samples were excited at 405 nm (3.06 eV).

In solution, the slower fluorescence decay of externally bound ThT can be explained by a change of the microenvironment around the dye molecules. ThT has been found to be a sensitive probe of DNA microviscosity.^36^ The internal rotation of ThT rings is hindered to some extent by the increase of the local microviscosity at the external binding sites in comparison to the viscosity of the solvent (water). The three-exponential fitting of the fluorescence decay recorded for ThT in ssDNA indicates that there is also a distribution of emissive species at the binding sites. The most plausible explanation is that ThT adopts various structural configurations because of microviscosity gradients and DNA strand conformational microheterogeneity.^37^ Notably, the average fluorescence lifetime in the film (0.49 ns) is only slightly longer than that in the solution (0.39 ns). At the same time, fluorescence decays of ThT bound to ssDNA in both film and solution are much slower than the fluorescence decay of pristine ThT in the solid film (Figure 3 and Table 1). This indicates that the intramolecular rotation of ThT is already hampered by binding to the DNA strand to such an extent that the solidification of the sample exerts only a minor effect.

**Table 1:** Fluorescence Decay Times (τ_i_) of ThT-Stained DNA Structures in Solutions and Drop-Cast Films Obtained by Fitting Three-Exponential Functions to Fluorescence Decays^a^

	solution				drop-cast film			
	τ_1_ (ns)	τ_2_ (ns)	τ_3_ (ns)	τ_avg_ (ns)	τ_1_ (ns)	τ_2_ (ns)	τ_3_ (ns)	τ_avg_ (ns)
ThT	0.009 ± 0.002 (0.999)	1.1 ± 0.01 (0.001)			0.11 ± 0.01 (0.94)	1.29 ± 0.01 (0.06)		
calf thymus DNA	0.14 ± 0.01 (0.74)	0.70 ± 0.01 (0.21)	2.37 ± 0.01 (0.05)	0.37	0.20 ± 0.01 (0.75)	0.88 ± 0.01 (0.21)	2.62 ± 0.02 (0.04)	0.44
ssDNA	0.15 ± 0.01 (0.66)	0.65 ± 0.01 (0.29)	2.09 ± 0.01 (0.05)	0.39	0.23 ± 0.01 (0.63)	0.82 ± 0.01 (0.33)	1.96 ± 0.01 (0.04)	0.49
dsDNA with internal loop	0.21 ± 0.01 (0.54)	0.93 ± 0.01 (0.34)	2.41 ± 0.01 (0.12)	0.72	0.25 ± 0.01 (0.61)	0.90 ± 0.01 (0.33)	2.04 ± 0.02 (0.06)	0.57
G-quadruplex	0.37 ± 0.01 (0.36)	1.39 ± 0.02 (0.40)	3.23 ± 0.01 (0.23)	1.43	0.38 ± 0.01 (0.55)	1.28 ± 0.01 (0.39)	2.94 ± 0.03 (0.06)	0.88

a Deconvolution of the experimentally measured IRF was taken into account as described in the Supporting Information. τ_avg_ is the amplitude-weighted average fluorescence lifetime.

Fluorescence decays recorded in liquid and solid phases were compared also for ThT mixtures with other studied DNA types (Figure S3). The result for ctDNA is very similar to that for ssDNA: the evaporation of water slows the relaxation down because of additional immobilization of externally bound ThT molecules in the solid state. This effect is in agreement with the postulated above mechanism involving FRET between intercalated and externally bound dye molecules. In the case of efficient FRET, as postulated for ctDNA, the fluorescence lifetime of the short-lived energy acceptor (externally bound molecules) determines the fluorescence kinetics of the whole system. The lack of long-lived energy donor (intercalated molecules) in ssDNA only weakly affects the fluorescence decays of the energy acceptor. Thus, the immobilization of externally bound molecules similarly extends the average fluorescence lifetime of ThT with both types of DNA, even though there are no intercalated ThT species in ssDNA.

The opposite, and actually surprising, effect is seen for mixtures of ThT with dsDNA and G4 DNA. Fluorescence decays become significantly faster after evaporation of water from the samples; therefore, the change cannot be attributed to immobilization of the dye in the solid environment. Effects such as the increased rigidity of the dried medium, reduced free volume available for ThT molecules, and steric hindrance in a more crowded environment should rather lead to the increase, not to the observed decrease of the fluorescence lifetime. Thus, in this case, the change of the fluorescence kinetics results most probably from the change of the preferred binding mode and distortion of the DNA cavity due to water evaporation.

Evaporation of strongly polar water molecules lowers the dielectric constant of the medium. It weakens the π-stacking interactions of DNA bases which may cause helix melting if the DNA sequence is too short.^38^ However, it is not the case in the studied DNA samples whereby water evaporation does not cause significant shortening of ThT lifetime, suggesting that ctDNA and DNA oligonucleotides 28 bases long retain their secondary structures and no denaturation occurs. However, water evaporation can make the tertiary DNA structure vulnerable to changes of the dielectric constant, and the internal loop in the dsDNA as well as G4 DNA can be perturbed, which can cause the loss of the emissive ThT species inserted in the cavities. The loss of the emissive species is manifested by the reduced contribution of the long-lived components to the fluorescence decays.

On the other hand, lowering of the dielectric constant strengthens electrostatic interactions between ThT and DNA phosphate groups responsible for the external binding. Therefore, it should be expected that water evaporation shifts the preference for the binding mode toward the latter, at the expense of binding inside DNA cavities. In agreement with the proposed mechanism, in dried samples the contribution of the short-lived fluorescence components increases and the contribution of the long-lived ones decreases.

In summary, time-resolved fluorescence measurements confirm that ThT can bind to DNA in a structure-specific mode in DNA cavities as well as in two nonspecific binding modes: intercalation between the DNA strands and external binding to the DNA phosphate groups. The external binding to the DNA helix is preferred over binding in cavities in the solid state, and the opposite is true in solutions of cavity-containing DNA.

Transferring ThT-stained DNA samples into the solid state allows studying bioderived films using the amplified spontaneous emission (ASE) effect. The fundamental principle that underlies generation of ASE is multiplication of photons in the stimulated emission process. In a medium with the population inversion (*i.e.,* in which more molecules occupy the electronic excited state than the ground state) the spontaneously emitted light is amplified as it propagates through the medium, which leads to the directional emission of light with its spectrum significantly narrower than that of fluorescence. ASE cannot be generated in pristine ThT, which undergoes ultrafast relaxation and exhibits very weak fluorescence. Thus, only ThT molecules immobilized because of DNA binding contribute to light amplification and the generation of ASE.

ASE was investigated in the ThT-stained DNA samples using the experimental setup depicted in Figure S1 by the gradual increase of the excitation energy with a simultaneous observation of the emission spectrum and the intensity of the emitted light (Figures 4 and S4). A rapid increase of the emission intensity and narrowing of the spectrum indicate that the spontaneously emitted fluorescence is amplified into ASE (Figure 4d). The excitation intensity at which this happens is the ASE threshold (inset in Figure S1).

**Figure 4:**
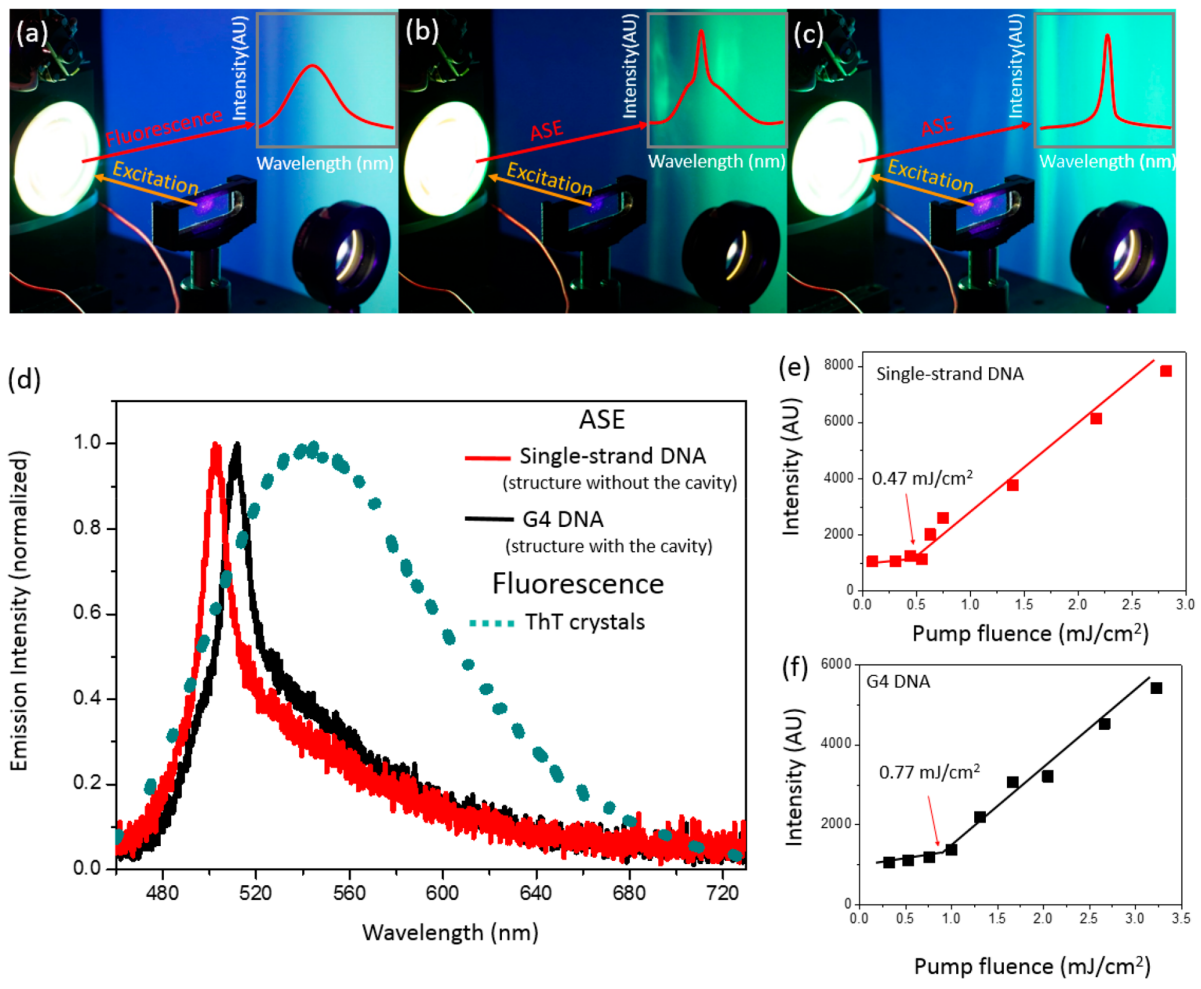
(a) Fluorescence and (b and c) amplified spontaneous emission (ASE) visualized in a real-time experiment. (d) Fluorescence (dashed) and ASE (solid lines) spectra in films prepared of ThT-stained single-strand DNA and G4 DNA. (e and f) Emission/excitation intensity dependences used for determination of the ASE thresholds for ThT in the presence of single-strand DNA and G4 DNA. ThT concentration in the solution before drop casting was equal to 45 μM in each case.

In the case of the ThT-stained DNA samples it was found that the ASE spectrum, width, and threshold depend on the arrangement of dye molecules in relation to the specific DNA structure. As a result, the spectral position of the ASE maximum could shift between 501 and 512 nm; fwhm was determined to be between 12 and 21 nm at a fixed excitation energy of 1.5 mJ/cm^2^, and ASE thresholds varied between 0.21 and 0.77 mJ/cm^2^ (Figure 4e,f).

Unexpectedly, the ASE threshold is higher with a longer ThT fluorescence lifetime. Whereas it could be expected that the longer fluorescence lifetime corresponds to a less efficient nonradiative deactivation, thus more favorable conditions for the ASE generation and a lower ASE threshold, this is not the case. The explanation of this surprising effect may be indicated by the fact that the lowest ASE threshold, much lower than for the other studied DNA types and more than twice lower than for ssDNA, is observed for ctDNA. This is the only DNA type among the studied ones that allows ThT intercalation in a long double helix. As has been observed by the super-resolution microscopy, intercalated molecules are aligned with respect to each other and exhibit a high orientational order.^39^ In terms of ASE generation, it means an alignment of electronic transition dipole moments and a higher probability of stimulated emission processes than in a system of randomly oriented molecules; the probability of inducing a stimulated emission act in one molecule by a photon spontaneously emitted by another molecule is the highest for molecules with mutually parallel transition moments. Thus, the lowest ASE threshold measured for ThT with ctDNA may reflect the local order of intercalated dye molecules.

On the other hand, the highest ASE thresholds, significantly higher than for ssDNA, are observed for both cavity-containing DNA types (Table 2). As discussed above, for both dsDNA and G4 DNA the fluorescence lifetime decreases after solidification of liquid samples, which probably reflects distortion of the cavities. The same mechanism may be responsible for the relatively high ASE thresholds.

**Table 2:** Positions of the ASE Maxima, ASE Spectrum Widths, and ASE Thresholds Determined for Drop-Cast Films of Various Types of ThT-Stained DNA^a^

	ASE wavelength maximum (nm)	ASE spectrum fwhm (nm)	ASE threshold (mJ/cm^2^)
ctDNA	509	14	0.21 ± 0.05
ssDNA	506	17	0.47 ± 0.02
dsDNA with internal loop	501	12	0.62 ± 0.06
G-quadruplex	512	20	0.77 ± 0.07

a For each sample, ThT concentration in the solution before drop casting was equal to 45 μM.

The presented ASE experiments carried out with various DNA types suggest that ASE thresholds reflect the DNA structure and the preferred binding mode in the solid state.

In summary, we explored ThT interactions with four DNA molecules: calf thymus DNA duplex, single-stranded DNA, double-stranded mismatched DNA with the internal loop, and G-quadruplex DNA. ThT-stained samples were investigated in solutions and in drop-cast dried films. Polarized light spectroscopy, time-resolved fluorescence, and amplified spontaneous emission measurements revealed that ThT interacts with DNA in two nonspecific binding modes, intercalation and external binding, and one structure-specific binding in DNA cavities. The interplay between the three binding modes determines the effective fluorescence quantum yield of ThT in relation to the specific DNA structure. Additionally, the drop-cast films were analyzed using the amplified spontaneous emission (ASE) technique. The ASE threshold was shown to be a sensitive parameter to the specific DNA structure. This indicates that ThT, which is a histological dye, can be easily utilized in ASE-based detection in medical applications, for example in cancer therapies where various DNA structures can be found.^40^

## Supporting Information Available

Materials, preparation protocols, and methods descriptions; fluorescence spectra of ThT in the presence of different DNA structures; lifetime decays, ASE spectra, and thresholds (PDF)
