# Supplementary material for: Identification of Thioflavin T Binding Modes to DNA: A Structure-Specific Molecular Probe for Lasing Applications
Source: J Phys Chem Lett. 2021 Jun 3;12(22):5436–42. doi: 10.1021/acs.jpclett.1c01254 (PMC8280760; doi:10.1021/acs.jpclett.1c01254)
Supplement: Supplementary file 1 — jz1c01254_si_001.pdf [file jz1c01254_si_001.pdf]

SUPPORTING INFORMATION

Identification of Thioflavin T Binding Modes to DNA: a Structure-Specific Molecular Probe for Lasing Applications

P. Hanczyc[1*], P. Rajchel-Mieldzioc[1], B. Feng[2], P. Fita[1]

[1]Institute of Experimental Physics, Faculty of Physics, University of Warsaw, Pasteura 5, 02-093 Warsaw, Poland

[2]Department of Chemistry and Chemical Engineering, Chalmers University of Technology, 412 96 Gothenburg, Sweden

Materials and methods

Thioflavin T: Ultrapure grade Thioflavin T (ThT) was purchased from AATBioquest. A stock solution was prepared in 1x PBS buffer (1x PBS is a phosphate-buffered Saline containing NaCl: 137 mM, KCl: 2.7 mM, Na_2HPO_4: 10 mM, KH_2PO_4: 1.8 mM) at a concentration of 0.5 mM. The concentrations of ThT solutions were assessed on the basis of absorbance at 412 nm with the molar extinction coefficient assumed as 36,000 $M^{-1}cm^{-1}$.[1]

Double-stranded calf thymus DNA (type I) (named ctDNA duplex in the text) was purchased from Sigma Aldrich. ctDNA was dissolved in 1x PBS buffer to the final concentration of 3.03 mM (1 mg/ml). The concentrations of ctDNA solutions were assessed per average mass of a single base (330 g/mol).

The following oligonucleotides were purchased from FutureSynthesis (Poland) and used as obtained. All samples were dissolved in 1x PBS.

Single-stranded DNA (ssDNA):

5'- GTTA AACT GCCT CTAC CCAT GCAT GGCC -3'

The concentrations of ssDNA solutions were assessed on the basis of absorbance at 258 nm with the molar extinction coefficient of 254500 M^{-1} cm^{-1} and the sequence molecular weight (M_w) of 8564 g/mol. The stock solution of ssDNA was adjusted to 115 µM (1mg/ml).

double stranded mismatched DNA (mismatched dsDNA with the internal loop)**:**

5'- GTTA AACT GCCT CTAC CCAT GCAT GGCC -3'

5'- GGCC ATGC ATGG CATC AGGC AGTT TAAC -3'

The concentrations of mismatched dsDNA solutions were assessed on the basis of absorbance at 258 nm with the molar extinction coefficient of 522700 M^{-1} cm^{-1} and the sequence molecular weight (M_w) of 17239 g/mol. The stock solution was prepared by dissolving single strands DNA in equimolar concentrations so that the final concentration was 57 µM (1mg/ml). DNA was hybridized by rising the temperature to 95°C and then slowly cooling down to room temperature in order to form the mismatched duplex.

G-quadruplex (G4 DNA):

5'- GGGG TTTT GGGG TTTT GGGG TTTT GGGG -3'

The concentrations of G4 DNA solutions were assessed on the basis of absorbance at 258 nm with the molar extinction coefficient of 262000 M^{-1} cm^{-1} and the sequence molecular weight (M_w) of 8935 g/mol. The stock solution concentration was equal to 110 µM (1mg/ml). The G-qudruplex sequence was kept at 95°C for 3 minutes and then slowly cooled down to room temperature in order to form the G4 structure.

ThT-stained solutions were prepared by mixing [dye]/[DNA base] in ratio from 0.00067 to 0.04. In order to obtain film samples the solutions were drop casted on glass slides in volume 100 µl and allowed to dry in ambient conditions.

UV-Vis Spectroscopy: Absorption spectra were recorded on a CARY-5000 spectrophotometer. Linear dichroism (LD) spectra were measured using Chirascan CD spectrophotometer. LD is defined as the difference in absorbance of light linearly polarized parallel ($A_{\parallel}$) and perpendicular ($A_{\perp}$) to the orientation axis of the DNA. Orientation was achieved using unit flow Couette cell. The sample was shear aligned in 0.5 mm space between two concentric quartz cylinders in a custom made outer-rotating Couette cell under a shear gradient of 3100 s^{-1}. The total path length of the Couette cell was 1 mm. All spectra were corrected for background contributions by subtracting corresponding spectra recorded without shear/rotation.

$$LD = A_{\parallel} - A_{\perp} \ (1)$$

The reduced *LD* (LD^r) is obtained by dividing the *LD* by the absorbance of the corresponding isotropic sample (A_{iso}), which for an anisotropic sample can be obtained as $A_{iso} = (A_{\parallel}+2A_{\perp})/3$, so that

$$LD^r = \frac{LD}{A_{iso}} = \frac{3LD}{A_{\parallel} + 2A_{\perp}} \ (2)$$

For molecules with a uniaxial orientation distribution, as can be expected for DNA in a Coutte cell, LD^r is a product of an orientation factor *S* and an optical factor *O*.

$$LD^r = S \, x \, O = \frac{3}{2} S\left(3\cos^2\alpha - 1\right) \ (3)$$

The optical factor *O* is related to the angle α between the helix axis and the light absorbing transition moment of the actual chromophore, which could be a DNA base or a DNA-bound ligand. The orientation factor $S = 0.5\,(3\langle\cos^2\theta\rangle - 1)$ corresponds to the average orientation of the DNA helix in the flow solution, where θ is the angle between the macroscopic orientation direction and the local helix axis of a particular molecule and where the average runs over all DNA molecules in the sample. The degree of helix orientation *S* was calculated from the LD^r values at 260 nm using the average angle $\alpha_{DNA} = 86^o$ for the DNA bases. The binding angle α_{dye} for a dye with respect to the DNA helix axis can then be calculated from the LD^r value in the visible region of its absorption spectra.

Steady-state fluorescence spectroscopy: For recording fluorescence spectra samples were excited with the light of a xenon arc lamp passing through a monochromator with the central wavelength and transmission bandwidth set to 400 nm and 1 nm, respectively, and a short-pass absorption filter (absorption edge at 450 nm). The emitted light was analysed with a SpectraPro 150 Czerny-Turner imaging monochromator equipped with a CCD camera (Andor DU420A-BU2). The fluorescence light was collected at the right angle to the direction of the excitation light. The cuvette with the solution was oriented at 90° and the films were oriented at approx. 45° angle with respect to the excitation and detection directions. A long-pass filter (edge at 430 nm) in front of the monochromator was used to eliminate the scattered excitation light. The recorded spectra were corrected for the transmission curve of this filter. The overall

spectral responsivity curve of the apparatus was determined using secondary emission standards according to the procedure proposed by Gardecki and Maroncelli[2].

Time-resolved fluorescence. Fluorescence decays were recorded using a custom-built setup based on the PicoQuant HydraHarp 400 Multichannel Picosecond Event Timer and the picosecond diode laser PicoQuant LDH-P-C-405B working at 405 nm. Repetition frequency of the laser was set to 10 MHz and the average excitation power was in the range 0.5 µW to 2.5 µW. Liquid samples were oriented at 90° and film samples were oriented at approx. 45° with respect to the excitation beam, with the fluorescence light collected at the approximately right angle with respect to the direction of the excitation beam. The collected light passed through a long-pass cut-off filter in order to remove the scattered excitation light. As the setup was used for measurements of liquid samples, the fluorescence light passed through a polarizer, whose transmission axis was oriented at the magic angle (54.7°) with respect to the polarization of the excitation beam, in order to eliminate artifacts due to the rotational diffusion. The fluorescence light was focused on the entrance slit of a Czerny-Turner monochromator (Acton Research SpectraPro 150) used to select a given wavelength from the fluorescence spectrum. After the monochromator the light was detected with the Hamamatsu R3809U-50 photomultiplier sensitive in the 160-850 nm range. The output signal from the photomultiplier was amplified with the Ortec Model 9327 amplifier prior to routing it to the HydraHarp module. The overall instrumental response function (IRF), measured by scattering the excitation light in a suspension of titanium dioxide, was narrower than 100 ps FWHM. Recorded fluorescence kinetics were analyzed by fitting with multiexponential decay functions convoluted with the IRF, using the Horiba FelixGX software. The average fluorescence lifetime was calculated as the amplitude-weighted mean of the decay times of each component.

Amplified spontaneous emission (ASE). Spectra of amplified spontaneous emission of ThT mixed with DNA structures in films were recorded in the experimental setup shown in Fig. S1.

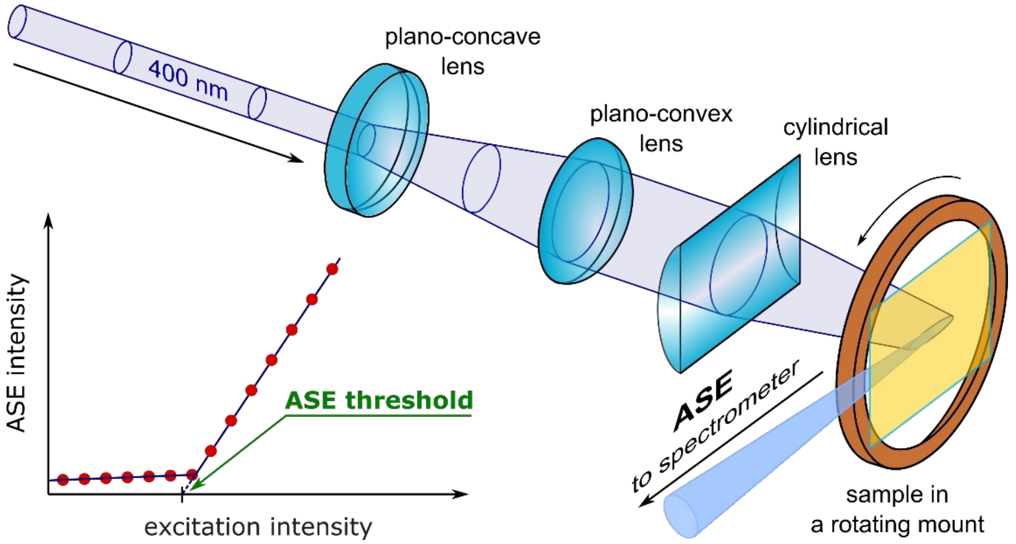

Fig. S1. Schematic view of the experimental setup used for studies of the ASE generation in drop-casted film samples. The telescope composed of the two spherical lenses followed by the cylindrical lens forms a horizontally elongated spot on the surface of the sample. The inset graph demonstrates the data analysis method used for the ASE threshold determination.

Studied films were excited at 400 nm with femtosecond pulses obtained by frequency doubling the output beam of a Ti:Sapphire femtosecond amplifier system (Legend Elite Duo) working at 5 kHz repetition rate. A beta-barium borate (BBO) crystal 1 mm thick was used to generate the second harmonic (SH) light of the amplifier output beam. Energy of the resulting SH pulses was adjusted by rotating the half-wave plate mounted in front of the BBO crystal. The SH light was separated from the remaining fundamental light by a pair of dichroic mirrors. The maximum energy of an SH pulse achievable in this configuration was approximately 300 μJ. After the dichroic mirrors the beam was first expanded with a telescope formed by a pair of spherical lenses with focal lengths of -50 and 125 mm. Then it was focused with a cylindrical lens in order to form a narrow stripe of light at the position of the film sample. The profile of the beam in the focal point was determined with the Thorlabs LC1-USB line CCD camera. It was approximately Gaussian in both directions with the full width at half maximum (FWHM) of (7.7 ± 0.3) mm in the horizontal and (17 ± 1) μm in the vertical direction, respectively. The area of the focal point at the FWHM intensity level, used for the calculation of average intensities of the excitation light reported in the current work, was taken as (0.0013 ± 0.0001) cm^2.

The studied films were oriented perpendicularly to the direction of the excitation beam. The amplified spontaneous emission was detected in the plane of the films, along the direction of the elongated linear focus of the cylindrical lens. The ASE light was collected with a fiber and delivered to the Ocean Optics USB 2000 spectrometer, whose spectral resolution was around 4 nm. For the determination of ASE thresholds the intensity of the excitation light was gradually increased by rotating the half-wave plate and the spectrum of the emitted light was simultaneously monitored. Plots of the dependence of the light intensity at the wavelength corresponding to the maximum of the ASE spectrum on the excitation intensity were used to determine the ASE generation thresholds.

Additional graphs

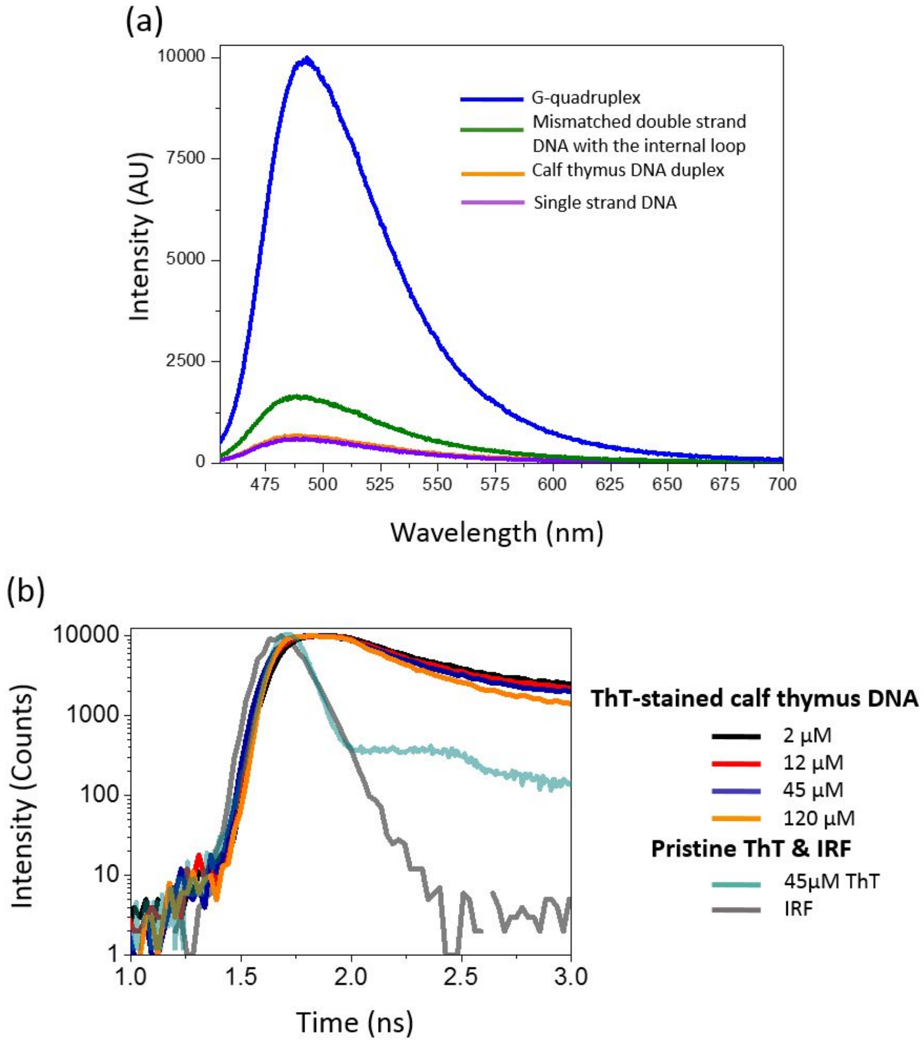

Fig. S2. (a) Steady-state fluorescence spectra of ThT-stained DNA in solution samples, (b) Examples of fluorescence decays in time range from 1 to 3 ns of ctDNA-ThT mixtures recorded at various ThT concentrations, 2 µM (black), 12 µM (red), 45 µM (dark blue) and 120 µM (orange), and of pristine ThT in a PBS solution (cyan). Samples were excited at 405 nm (3.06 eV). The instrumental response function (IRF) is shown in grey.

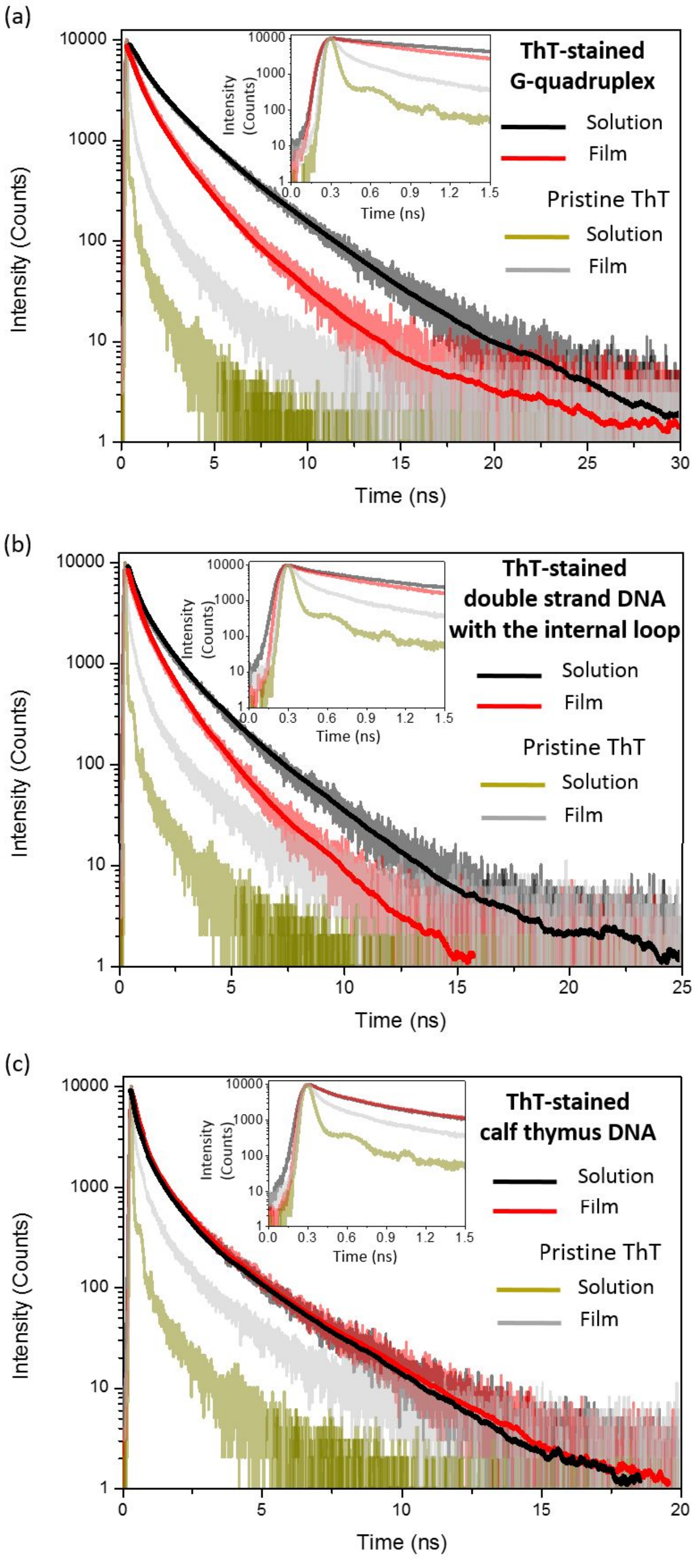

Fig. S3. Fluorescence decays of ThT-stained DNA samples in solutions and in drop-casted films: (a) G-quadruplex DNA, (b) mismatched double-strand DNA with the internal loop, (c) calf thymus DNA. Fluorescence decays of pristine ThT dissolved in the PBS buffer and drop-casted on a glass slide are included in each graph for the sake of comparison with DNA samples stained with ThT. The insets show decays in time scale to 1.5 ns.

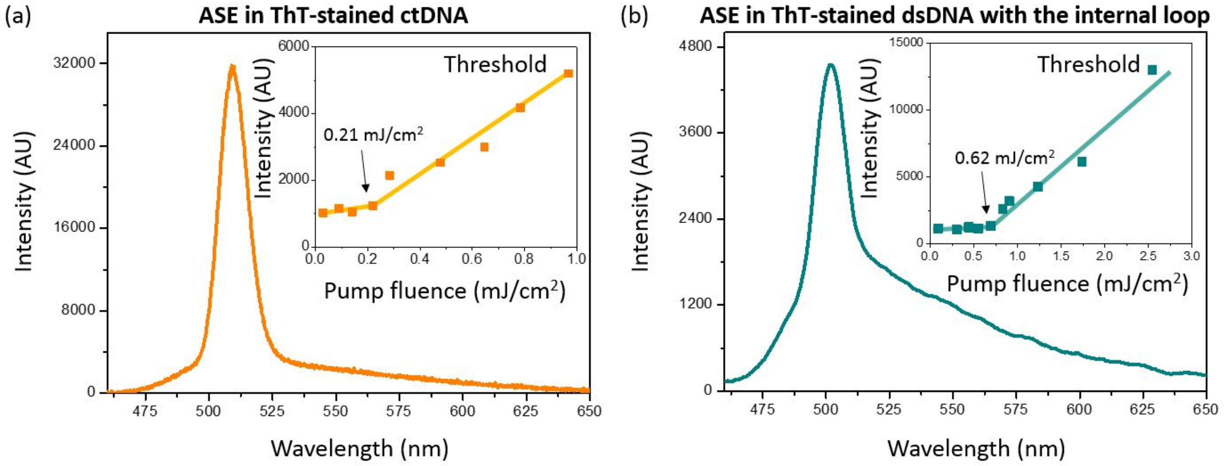

Fig. S4. ASE spectra in ThT-stained DNA samples: (a) calf thymus DNA, (b) dsDNA with the internal loop. The insets represent the ASE thresholds.

1. Groenning, M., Binding mode of Thioflavin T and other molecular probes in the context of amyloid fibrils—current status. *J. Chem. Biol.* **2010,** *3*, 1-18.
2. Gardecki, J.; Maroncelli, M., Set of secondary emission standards for calibration of the spectral responsivity in emission spectroscopy. *Appl. Spectrosc.* **1998,** *52*, 1179-1189.
